# Exogenous melatonin boosts vaccine-induced immunity in individuals with high pre-existing influenza immunity

**DOI:** 10.3389/fimmu.2025.1663763

**Published:** 2025-10-24

**Authors:** Kyosuke Oda, Janine Danko, Eileen Villasante, Elke Bergmann-Leitner, Rachel Lee, Wathsala Wijayalath

**Affiliations:** ^1^ Henry M Jackson Foundation for Advancement of Military Medicine, Inc., Bethesda, MD, United States; ^2^ Agile Vaccines and Therapeutics Department, Naval Medical Research Command (NMRC), Silver Spring, Maryland, MD, United States; ^3^ Department of Translational and Clinical Research, Naval Medical Research Command, Silver Spring, MD, United States; ^4^ Biologics Research & Development, Walter Reed Army Institute of Research (WRAIR), Silver Spring, MD, United States; ^5^ Walter Reed National Military Medical Center, Bethesda, MD, United States; ^6^ Uniformed Services University of the Health Sciences, Bethesda, MD, United States; ^7^ Division of Allergy & Immunology, Department of Medicine, University of California, San Diego, San Diego, CA, United States

**Keywords:** melatonin, adjuvant, influenza vaccine, immunity, sleep, T follicular helper cells, immunoprofiling, immunomodulation

## Abstract

**Introduction:**

Naturally produced melatonin acts as an antioxidant and immunomodulator, regulating sleep and vital functions. Synthetic melatonin is widely used as a sleep aid by the general population, including U.S. military personnel. Immunomodulatory effects of melatonin on vaccines and therapeutics must be studied to develop and implement effective clinical practice guidelines, which will enhance the quality of life of the public and the military readiness. Here, we evaluated exogenous melatonin mediated immune modulation during seasonal influenza vaccination using the samples generated in the Melatonin and Vaccine Response, Immunity, and Chronobiology Study (MAVRICS) conducted by the Naval Medical Research Command (NMRC) and the Walter Reed National Military Medical Center (WRNMMC).

**Methods:**

MAVRICS participants had received quadrivalent inactivated influenza vaccine (IIV4) (2022/23 season) after being randomized to melatonin (REMfresh® 5mg melatonin caplets one hour before the planned bedtime for 14 days, starting on the night of vaccination) or no treatment (control). The hemagglutination inhibition (HAI) antibody responses, serum cytokine/chemokines, and *in vitro* antigen-specific cellular responses were measured at 24-48h pre-vaccination and 14–21 days post-vaccination. Peripheral blood mononuclear cells were stimulated with recombinant hemagglutinin proteins in vitro to measure antigen-specific responses. For the data analysis, participants were stratified by the baseline HAI titers of the A/Victoria vaccine strain.

**Results:**

Vaccination induced a significant increase in HAI antibodies, antigen specific circulating T follicular helper 17 (cTfh17) cells and IL-2, IL-4, IL-17A, IL-13 cytokines in the melatonin recipients who had high HAI baseline titers. These changes were not seen in their control counterparts. The cTfh17 levels remained unchanged and present at consistently high levels in the low HAI baseline melatonin recipients, while both cTfh2 and cTfh17 subsets were increased in those of the control vaccinees. Notably, melatonin itself did not significantly impact the global cytokine milieu in the serum.

**Discussion:**

The data suggest that the melatonin has a selective modulatory effect on the antigen-specific cTfh subset response based on the levels of pre-existing HAI antibodies and the previously imprinted immune landscape. Given the disease’s complex immune history, melatonin shows promise as a potential adjuvant for seasonal influenza vaccines.

## 1 Introduction

Melatonin (N-acetyl-5-methoxytryptamine) is a multifunctional indolamine synthesized by various organs in the body. The pineal gland produces approximately 5% of the body’s melatonin in response to light-dark cycle-related photic information (1). The photosensitive retinal ganglion cells in the eyes receive the photic signals and transmit them to the suprachiasmatic nucleus (SCN) located in the hypothalamus regulating the pineal melatonin secretion (2). Melatonin is also produced by many other organs such as brain, kidneys, liver, gastrointestinal tract, adrenal glands, heart, thymus, genital glands, placenta, uterus, platelets, and various immune cells, irrespective of the changes in the daylight (3). At the tissue level, these organs synthesize 10 to 400 times more melatonin via cellular mitochondria (4). Pineal-derived melatonin is released into both the bloodstream and cerebrospinal fluid (1), remaining largely separated from melatonin produced by other tissues (1). Acting as an endocrine messenger, pineal melatonin modulates various physiological processes and functions. The majority of these processes are associated with sleep, circadian rhythms, aging, metabolism, growth, thyroid hormone production, reproduction, circadian and seasonal changes in immunity, and the immune-pineal axis (3). Organ-derived melatonin functions as autocrine or paracrine molecules, most likely acting at the site of synthesis (1, 3). At the organ or tissue level, melatonin plays a crucial role as an antioxidant and an immunomodulator through multiple different mechanisms (1, 5). Functional versatility of melatonin could be attributed to its intrinsic ability to act through receptor-dependent or -independent manner and to cross all the morpho-physiological barriers (5).

Due to its antioxidant and immunomodulatory (6–9) properties, researchers have been intrigued by melatonin for decades as a potential treatment option for various communicable (10–15) or non-communicable diseases (16–19) or even as a vaccine adjuvant (20–27). This has prompted multiple experimental (12, 16, 17, 19, 25, 26) veterinary (15, 20–23), and clinical studies (10, 14, 28–30) to explore the impact of exogenous melatonin on the immune system under various inflammatory disease conditions and during vaccinations. Not surprisingly, melatonin’s influence on the immune system appears to be rather more complex and context specific, most likely depending on the baseline immunological status of the body. In fact, melatonin seems to exert both anti- and pro-inflammatory functions under different inflammatory and disease conditions (6, 9, 11, 29, 31–34). Anti-inflammatory actions of the melatonin have shown to be beneficial during acute viral or bacterial infections (31–34) and other chronic inflammatory disorders (9). Melatonin also seems to modulate antigen-specific T and B cells, cytokine responses, IgG antibodies and antigen presentation to T cells when tested as a vaccine adjuvant in mouse and veterinary research models (33, 35–39). The interactions of melatonin with various intracellular signaling pathways have also been reported under different disease conditions (40).

There is a complex bidirectional crosstalk between the central nervous system (CNS) and the immune system along the sleep-immune axis, affecting the outcome of a disease or a vaccination (41). For example, the immune system, mainly the leukocytes, can signal to the brain via neurotransmitters, neuropeptides (ex. cytokines and chemokines) and hormones upon encountering a foreign insult. As a result, the CNS signals trigger common symptoms such as fever, fatigue, and sleepiness potentially facilitating the recovery by saving energy and promoting the host defense. Sleep deprivation can induce chronic low-grade inflammation in the body causing chronic inflammatory disorders, leading to slow recovery from an infection and alter the efficacy of clinical vaccines. The potential importance of having proper sleep and administration of melatonin during vaccinations have been reiterated after the COVID-19 pandemic, particularly due to the individual variability in the efficacy of SARS-CoV-2 vaccination (42). In fact, several clinical studies have investigated the effect of sleep on the efficacy of human vaccines such as influenza, hepatitis A and hepatitis B (43). Some of these studies showed decreased anti-influenza antibody responses associated with sleep deprivation, short sleep duration or insomnia in the vaccinated individuals (43). Also, proper sleep after hepatitis A vaccination increased antigen-specific T helper 1 mediated cytokine responses and IgG1 antibody responses (43).

Among commonly approved clinical vaccines, the seasonal influenza vaccine faces yearly challenges due to various factors, resulting in significant variability in the baseline immunity and subsequent vaccine efficacy across the vaccine recipients. Challenges associated with aging, immunodeficiency, seasonal viral changes, and repeated exposure to infections or vaccinations are inherent and often unavoidable. While many intrinsic, extrinsic, environmental, behavioral, nutritional and vaccine factors can influence the outcome of vaccines, sleep is a common behavioral factor that can be rectified relatively easily without complex interventions (43). This is where melatonin could serve as a safer, all-in-one vaccine adjuvant with its sleep aid, immunomodulatory and antioxidant properties. Hypothetically, by promoting sleep following or during vaccinations, melatonin could modulate the baseline immune status and enhance the vaccine efficacy and durability while minimizing the adverse effects of the vaccine. Given that the clinical evidence to support the adjuvant effect of melatonin is limited, we sought to understand the effect of exogenous melatonin on cellular and humoral immune responses induced by 2022/23 seasonal influenza vaccination in the present study. Here, we used clinical samples generated by the “Melatonin and Vaccine Response, Immunity, and Chronobiology Study (MAVRICS)” to evaluate impact of melatonin on immune responses induced by 2022/23 influenza vaccine recipients. We established the immunological profile of vaccine-induced functional antibody responses (hemagglutination inhibition (HAI) titers), serum cytokines and antigen-specific T cells responses. The study participants were stratified by their baseline HAI titers to A/Victoria/2570/2019 (H1N1) to determine the impact of melatonin when taken for the first 14 days starting on the night of the vaccination. The results clearly demonstrate that melatonin was beneficial for individuals with a high HAI baseline, i.e., high pre-existing immunity.

## 2 Materials and methods

### 2.1 Summary of the clinical study design

The detailed clinical study design is published elsewhere (44). Briefly, the MAVRICS randomized, open-label pilot trial was conducted from October 2022 to January 2023 at the Walter Reed National Military Medical Center (WRNMMC) and the Naval Medical Research Command (NMRC) Clinical Trials Center in Bethesda, MD (Institutional Review Board approved under NMRC.2021.0006, clinicaltrials.gov NCT04953754). The goal of the study was to evaluate the impact of sleep, circadian health, and melatonin on vaccine immunogenicity and outcomes. Individuals who had received the influenza vaccine within 6 months prior to enrollment, or with any of the following conditions; an allergy or contraindication to influenza vaccine(s), medical history of an immune-compromising medical condition (i.e., HIV, active cancer, etc.), a diagnosed sleep disorder requiring medication (i.e., insomnia, narcolepsy, etc.), pregnancy, or recent history (within 3 months) of taking immunosuppressant or immune-modifying treatment(s) (i.e., systemic corticosteroids, chemotherapy, IVIG, blood transfusion, etc.), and/or use of sleep supplements or medications prescribed for sleep in the past month were excluded from the study. Healthy adults aged 18–64 years who were eligible and planning to receive influenza vaccination at WRNMMC were enrolled after eligibility/health screening and informed written consent. Participants were randomized to receive either melatonin (treatment) or no melatonin (control). The treatment group was given REMfresh^®^ 5mg caplets (Nestle Health Science), an over the counter (OTC) melatonin supplement, at a dose of a single caplet one hour before their planned bedtime for 14 days, starting on the night of influenza vaccination. The vaccine was administered between 8am and 4pm on the day of the vaccination. Both treatment and control group participants received quadrivalent inactivated influenza vaccine (IIV4) (FluLaval^®^ Quadrivalent, GSK, Quebec, Canada). The influenza strains included in the 2022/23 IIV4 vaccine included: A/Victoria/2570/2019 (H1N1) pdm09-like virus, A/Darwin/9/2021 (H3N2), B/Austria/1359417/2021 (B/Victoria lineage), and B/Phuket/3073/2013 (B/Yamagata lineage) (45). A schematic representation of our research study is provided in the **Supplementary Figure S1**.

### 2.2 Clinical samples

Blood samples were taken 24–48 hours (week 0) before and 14–21 days (2–3 weeks) (between 8am and 4pm) after the influenza vaccination for serum and to isolate peripheral blood mononuclear cells (PBMCs). Use of deidentified human clinical samples in this study was approved as non-human subject research through a Human Subject Research Determination conducted by the NMRC Institutional Review Board in compliance with all applicable federal regulations governing the protection of human subjects. The hemagglutination-inhibition (HAI) assay was conducted as a part of the clinical study.

### 2.3 Recombinant hemagglutinin proteins

The four Hemagglutinin (HA) proteins representing the 4 influenza strains included in the 2022/23 IIV4 vaccine were recombinantly produced by Sino Biological Inc., (Wayne, PA) and delivered to NMRC (cat# 40787-V08H1, 40859-V08H1, 40498-V08B, 40862-V08H). An additional lot of recombinant HA from A/Darwin/9/2021 (H3N2) strain was commercially purchased from eEnzyme (www.eenzyme.com -cat#IA-H3-D21WP). The recombinant proteins were used to stimulate cultured PBMCs to evaluate antigen-specific immune responses *in vitro.*

### 2.4 *In vitro* stimulation of PBMC cultures to assess frequency of antigen-specific T cell populations

As previously published, flow cytometric analysis of antigen-specific T cell populations was conducted by stimulating PBMC *in vitro* to induce upregulation of CD154 (i.e., early marker for antigen-specific T cell activation) (46–48). Briefly, paired pre- and post-vaccination PBMCs were thawed using complete RPMI 1640 media supplemented with 10% heat-inactivated human AB serum, Benzonase^®^ Nuclease (only for the initial thawing), 1x penicillin-streptomycin, 1x MEM non-essential amino acid solution, 1x L-Glutamine, 1x sodium pyruvate and 55mM 2-Mercaptoethanol (at 1:1000 final dilution). Three million total PBMCs from each sample were cultured in 96-well flat bottom plates (1.5 million cells/well) for 48h in presence of a cocktail containing the following components: mixture of HA recombinant proteins containing A/Victoria, A/Darwin, B/Austria, B/Phuket, each at 10 µg/mL final concentration, anti-human CD40 antibody at 1:50 dilution (CD40 pure – functional grade, human, clone#HB14, Miltenyi Biotec, Germany) and anti-human CD196 (CCR6)-APC at 1:10 dilution (clone# REA190, Miltenyi Biotec) per well. CD40 antibody was added to stabilize the expression of CD40 ligand (CD154). After 48h, culture supernatants were harvested for cytokine analysis by Meso Scale Discovery assay and stimulated PBMCs were harvested for antigen-specific CD154^+^ cell enrichment.

### 2.5 Identification of antigen-specific circulating T follicular helper subsets by flow-cytometry

**Figure 1:**
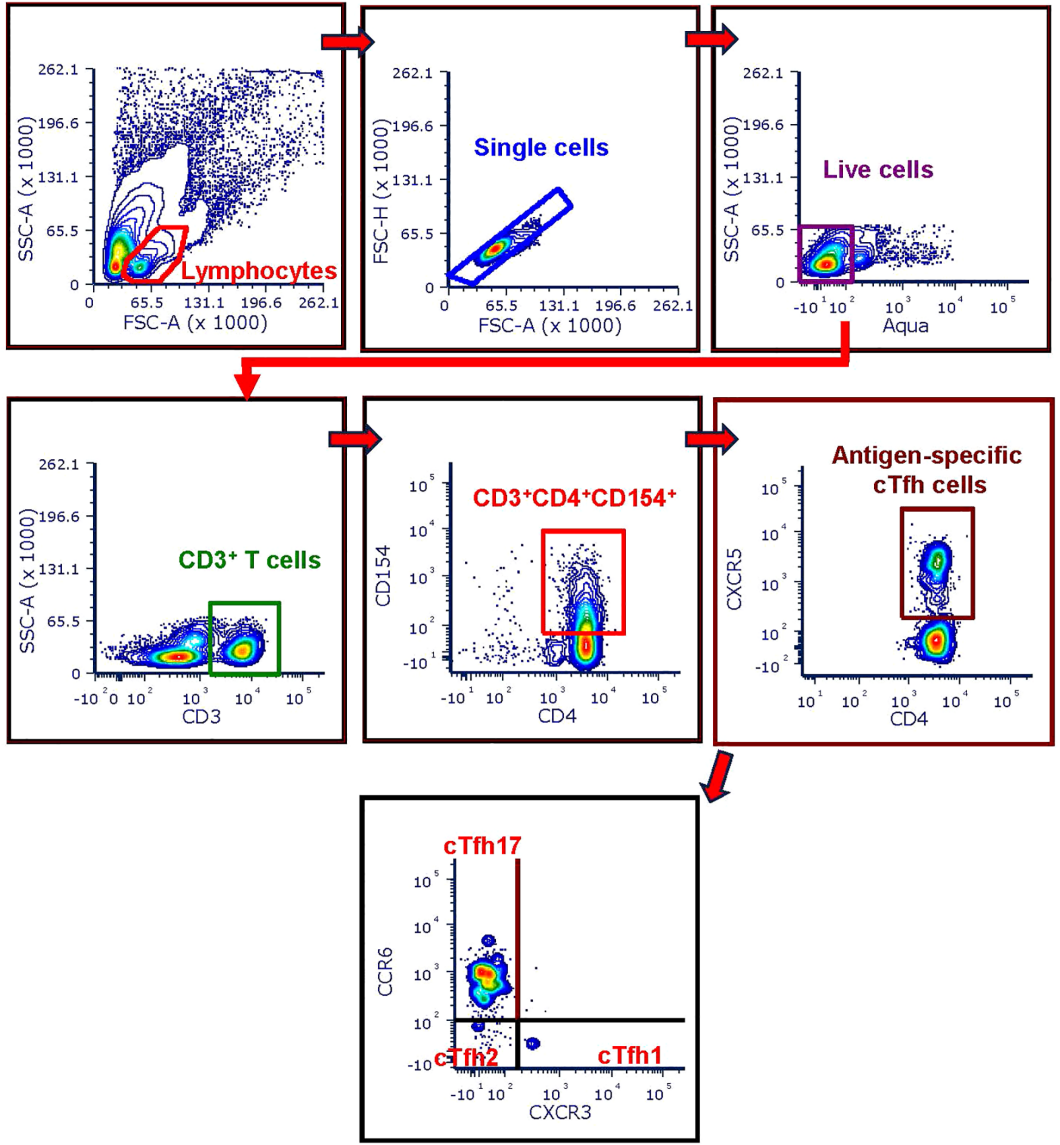
Flow-cytometry gating strategy used for cTfh analysis. CD154 enriched cells were first gated on CD3^+^ T cells after excluding doublets and dead cells from the parent lymphocyte population. Next, antigen-specific CD4^+^ T cells were identified as the CD3^+^ T cells co-expressing CD154 and CD4 surface markers (CD3^+^CD4^+^CD154^+^ T cells). Then the cTfh cells were identified as the CD3^+^CD4^+^CD154^+^ cells expressing CXCR5 (CD3^+^CD4^+^CD154^+^CXCR5^+^). The three cTfh cells subsets were further identified based on the expression of CXCR3 and CCR6 as follows: cTfh1 = CD3^+^CD4^+^CD154^+^CXCR5^+^CXCR3^+^CCR6^-^, cTfh2 = CD3^+^CD4^+^CD154^+^CXCR5^+^CXCR3^-^ CCR6^-^, cTfh17 = CD3^+^CD4^+^CD154^+^CXCR5^+^CXCR3^-^ CCR6^+^.

Stimulated PBMCs were first stained with Live/Dead Fix Aqua – 400 dye per manufacturer’s instructions (LIVE/DEAD™ Fixable Aqua Dead Cell Stain Kit, ThermoFisher Scientific, Waltham, MA). Next the cells were magnetically labelled with CD154-biotin antibody and anti-biotin microbeads per manufacturer’s instructions (Miltenyi Biotec). Subsequently the cells were washed with staining buffer containing 1x PBS, 1% BSA and 0.05% sodium azide and stained with a cocktail of anti-human surface antibodies for 45 min in presence of the FcBlock (Human TruStain FcX™, Biolegend, San Diego, CA). The surface antibody cocktail included the following antibodies (all obtained from Miltenyi Biotec): CD3-VioBlue (clone: BW264/56), CD4-PerCP-Vio700 (clone: M-T466), CD154-PE (clone: 5C8), CD185 (CXCR5)-PE-Vio770 (clone: REA103), CD183 (CXCR3) -VioBright FITC (clone: REA232). Stained cells were washed and magnetically enriched per manufacturer’s instructions (Miltenyi Biotec). The CD154^-^ cell fractions (flow-through of columns) were used to prepare crude protein lysates to analyze the expression of cell signaling factors. The CD154^+^ cells were acquired on a BD LSR Fortessa flow cytometer to identify the antigen-specific circulating T Follicular Helper (cTfh) cell subsets. The gating strategy used to analyze the phenotype and frequency of each immune cell subset is shown in the **Figure 1**. Data were analyzed using FCS express 7 (DeNovo Software).

### 2.6 Hemagglutination-inhibition assay

Influenza-specific serum antibodies were quantified by hemagglutination-inhibition (HAI) assays using standard procedures in blinded samples. Briefly, sera were treated at a 1:3 ratio (vol/vol) with receptor-destroying enzyme (RDE, Accurate Chemical & Scientific Corporation, New York, NY) at 37 °C for 18 to 20 hours to eliminate non-specific inhibitors of agglutination. RDE were subsequently inactivated by incubation at 56 °C for 45 minutes, followed by addition of 6 volumes of phosphate-buffered saline (PBS) resulting in an initial testing dilution of 1:10. All RDE-treated sera were tested for non-specific agglutinins, and positive sera were heme-adsorbed using turkey erythrocytes (LAMPIRE Biological Laboratories, Inc, Pipersville, PA) prior to performing the HAI assay; each sample was tested in duplicate (49). Influenza virus standardized to 8 hemagglutinin units (HAU) per 50 μL (4HAU per 25 μL) in PBS was added to two-fold serial dilutions of test sera. Following incubation at room temperature for 30 minutes, 0.5% turkey red blood cells were added. Plates were observed for agglutination after 30 minutes. The HAI titer was defined as the reciprocal of the highest dilution of serum that completely inhibited hemagglutination. The geometric mean titer (GMT) was calculated for each sample duplicate and reported as the final titer. For computational purposes, titers of <1:10 were assigned a value of 1:5. pre-and post-vaccination serum samples were processed and tested together (49).

### 2.7 Cytokine/chemokine analysis by mesoscale assays

Circulating cytokines and chemokines present in serum were analyzed by U-PLEX TGF-β Combo (human) (analytes; TGF-β1, TGF-β2, TGF-β3), V-PLEX Chemokine Panel 1 Human Kit (analytes: Eotaxin, Eotaxin-3, IL-8, IL-8 (HA), IP-10, MCP-1, MCP-4, MDC, MIP-1α, MIP-1β, TARC) and V-PLEX Proinflammatory Panel 1 Human Kit (analytes: IFN-γ, IL-1β, IL-2, IL-4, IL-6, IL-8, IL-10, IL-12p70, IL-13, TNF-α) per manufacturer’s instructions (Meso Scale Diagnostics (MSD) LLC, Rockville, MD). Antigen-specific cytokines present in the cell culture supernatants were analyzed by customized U-PLEX multiplexed mesoscale assay kits (U-PLEX Custom Biomarker Group 1 (human), MSD) per manufacturer’s instructions. The following cytokines were included in the U-plex panel: IL-2, IFN-γ, TNF-α IL-4, IL-5, IL-6, IL-8, IL-9, IL-10, IL-12p70, IL-13, IL-1α, IL-1β, IFN-β, IL-17A, IL-17/E/IL-25, IL-17F, IL-21, IL-22, IL-23, IL-27, IL-31, IL-33. Concentration of each cytokine (pg/mL) was determined using MESO QuickPlex SQ 120 instrument supported by the Discovery Workbench Software (MSD) per manufacturer’s instructions.

### 2.8 Cell signaling factors by MILLIPLEX ^®^ bead-based Luminex assay

A multiplexed cell signalling Luminex detection assay (MILLIPLEX^®^ 9-plex Multi-Pathway Signaling Magnetic Bead kit, EMD Millipore, Burlington, MA) was used to analyze the presence of intracellular cell signalling factors CREB, JNK NF-kB, P38, ERK, STAT 3, STAT 5, Akt and p70 S6 kinase (p706SK) proteins in 25 µg of the protein lysates (CD154 negative cells). To prepare the crude protein lysates from CD154 negative cells, the cells were washed once with ice cold 1x PBS, re-suspended in 100 µL of freshly prepared ice-cold 1X MILLIPLEX^®^ Lysis Buffer containing 1x protease inhibitor cocktail (ThermoFisher Scientific, Waltham, MA), vortexed vigorously for 30 seconds, and stored at -20^0^C. A bicinchoninic acid (BCA) assay (Pierce™ BCA Protein Assay Kits, ThermoFisher Scientific) was used per manufacturer’s instructions to determine the protein concentrations. A standard curve was generated by the GraphPad Prism, v10.1.2 software by fitting the Optical Density (OD) measurements and respective concentrations of the serial dilutions of the BCA standards to sigmoidal 4-PL model (GraphPad Prism, v10.1.2).

### 2.9 Statistical analysis

Appropriate statistical tests were applied for each comparison after determining the normality of the data by Shapiro-Wilk test and Kolmogorov-Smirnov tests at two-sided, 0.05 alpha level (GraphPad Prism, V10.1.2). R studio (Version 2023.12.1, Build 402,” Ocean Storm”) was used to generate correlograms (spearman correlation, two-sided, 0.05 alpha level) and Principal Component Analysis (PCA) plots.

## 3 Results

### 3.1 Melatonin modulates HAI antibody responses and antigen-specific cTfh cells following influenza vaccination.

Antibodies and Tfh cells play a key role during an effective antibody mediated immune response (50, 51). Neutralizing antibodies against HA protein prevent influenza virus from entering the host cells. HAI antibody titers are generally associated with reduced risk of influenza infection and protection induced by inactivated influenza vaccines (IIVs) (52). Several IIV studies have also reported associations between vaccine induced antibody responses and circulating subsets of CD4^+^ Tfh cells (53–55). Tfh cells promote the selection of high-affinity B cells in germinal centers, resulting in the expansion of memory B cells that produce high-affinity antibodies (51). Here, we evaluated whether the exogenous melatonin modulates these key immune mediators using serum and PBMCs collected from the IIV4 recipients who participated in the MAVRICS clinical trial.

**Figure 2:**
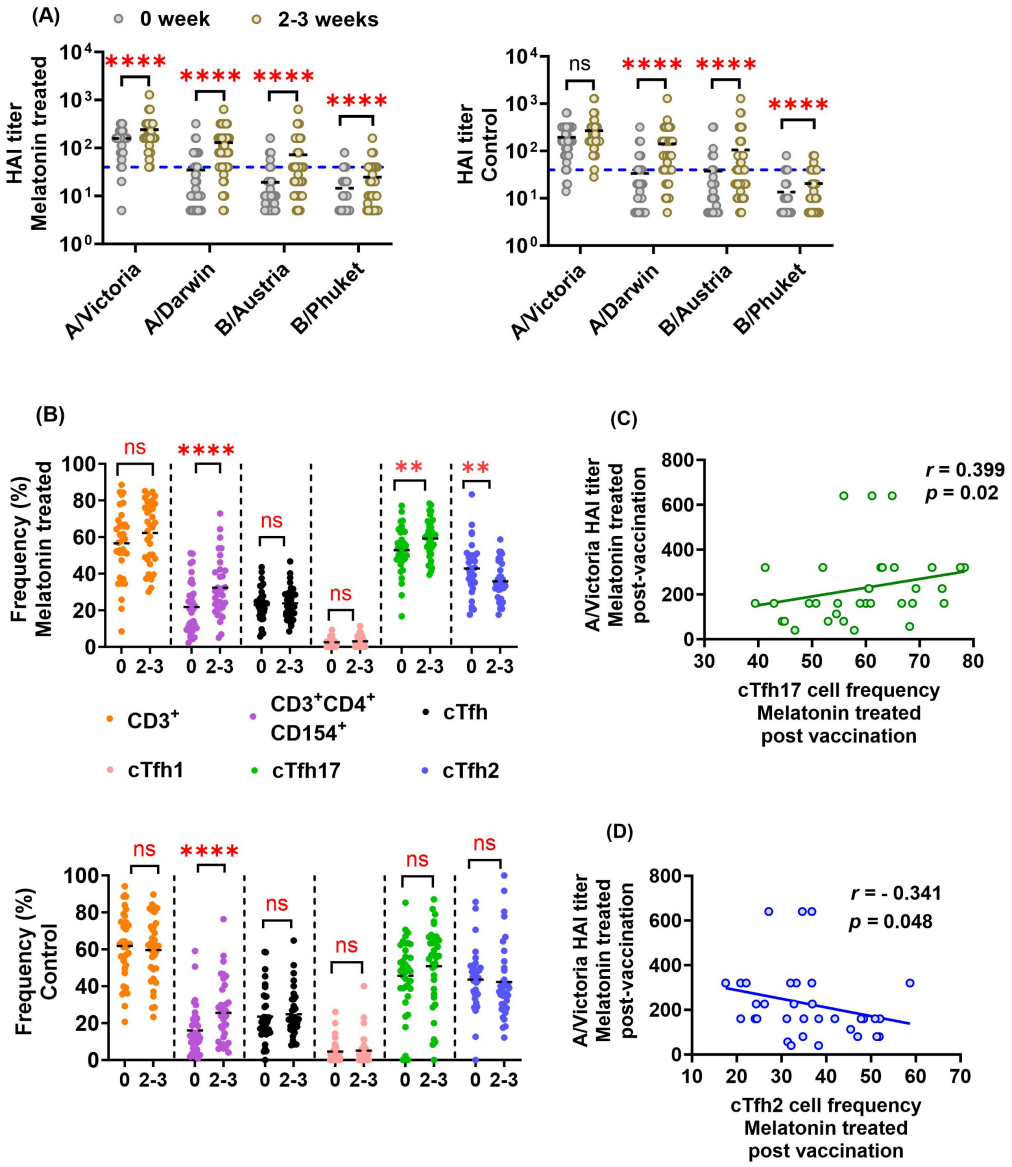
Effect of exogenous melatonin on HAI antibody titers and antigen-specific cTfh cell responses in IIV4 recipients. **(A)** HAI antibody titers against each of the four vaccine strains before (0) and at 2–3 weeks (2-3) in the melatonin recipients (n=53, left panel) and the control vaccinees (n=55, right panel). 1:40 HAI titer cut-off is indicated by the blue dashed line. **(B)** Frequency of HA antigen-specific T cell subsets in the melatonin recipients (n=34, top panel) and the control vaccinees (n=36, bottom panel) before (0) and 2–3 weeks (2-3) after IIV. The black dashed line represents the cohort mean. Asterisks (*) denote significant differences between pre- and post-vaccination at 0.05 alpha level. **P < 0.01; ****P < 0.0001; ns, not significant. Paired comparisons were carried out using either paired sample T test or the Wilcoxon matched pairs signed rank test after testing for normality. Spearman correlation between **(C)** A/Victoria HAI titers and antigen-specific cTfh17 frequency **(D)** A/Victoria HAI titers and antigen-specific cTfh2 frequency in the melatonin recipients 2–3 weeks post-vaccination. Correlation coefficient (*r)* was calculated using Spearman’s Rank Correlation test with an alpha level of 0.05.

Our overall analysis shows that the vaccinated individuals who received the melatonin treatment (n=53) had significantly higher HAI titers against each of the four vaccine strains after vaccination (**Figure 2A**). Likewise, the control vaccine recipients (n=55) had significantly elevated HAI titers against three of the vaccine strains: A/Darwin, B/Austria and B/Phuket (**Figure 2A**). However, the control cohort did not show a significant increase in A/Victoria HAI titers post-vaccination (**Figure 2A**). We also measured the frequency of Influenza-specific T cells in PBMCs stimulated *in vitro* with a mixture of recombinant HA proteins representing the four vaccine strains (**Figure 2B**). **Figure 2B** shows that the Influenza vaccination induced significantly higher levels of activated CD3^+^CD4^+^CD154^+^ T cells in both groups of vaccinees, 2–3 weeks after vaccination. In contrast, only melatonin recipients showed significantly elevated cTfh17 and reduced cTfh2 levels (**Figure 2B**, top panel). In addition, the melatonin-treated cohort showed a significant positive correlation between A/Victoria HAI titers and cTfh17 cell frequencies induced by the vaccination (**Figure 2C**). As expected, cTfh2 cell frequency was inversely correlated to the A/Victoria HAI titers in this group (**Figure 2D**). Notably, cTfh1 cell frequencies were relatively lower in both groups of vaccinees with an average frequency (± standard deviation) of 2.98 ± 2.98 in melatonin recipients and 5.08 ± 7.90 in control vaccinees (**Figure 2B**). Overall, the exogenous melatonin appears to impact HAI antibodies and antigen-specific cTfh17 and cTfh2 cells induced by IIV4 vaccination.

Baseline immune signatures can reshape the immune landscape generated by the influenza vaccination particularly due to previous infections, vaccinations, or age (56). After our preliminary data analysis, we noticed that the baseline HAI responses against A/Victoria/2570/2019 (H1N1, pdm09-like) strain were significantly higher compared to that of the three other vaccine strains in both cohorts (**Figure 2A**, P <0.0001, Friedman Test with Dunn’s correction for *post hoc* pairwise comparisons). Higher titers against the A/Victoria strain may be due to the higher prevalence of A/H1N1, pdm09-like strains over the past flu seasons (data since 2015–2016 season, FluView Interactive, Center for Disease Control and Prevention) (57), and previous exposures to H1N1 seasonal vaccinations. The majority of the participants had low baseline titers below 1:40 against the other vaccine strains, which is lower than the 50% seroprotective HAI titer threshold defined by the European Medicines Agency (EMA) committee for Medicinal Products for Human use (CHMP) and Food and Drug Administration (FDA), USA (58) (**Figure 2A** blue line for 1:40 titer cut-off). To account for the baseline differences on subsequent melatonin mediated vaccine induced immune responses, we stratified each vaccine cohort by their HAI baseline responses to the A/Victoria/2570/2019 (H1N1) influenza strain. Individuals with A/Victoria HAI baseline titers above 1:100 were herein classified as the high baseline group, while those with titers below 1:100 were classified as the low baseline group within each cohort. This 1:100 titer cut-off was set arbitrarily given about 96% of vaccine recipients from each group had A/Victoria baseline HAI titers ≥1:40 (**Figure 2A** blue line for 1:40 titer cut-off). Notably, the low HAI baseline group showed a higher seroconversion rate against the A/Victoria strain (25% - melatonin and 27.27% - control) compared to those in the high HAI baseline group (4.55% - melatonin and 4.35% -control) regardless of the treatment (**Table 1** high and low baseline). Likewise, A/Victoria HAI titer fold increase was significantly higher in the low baseline group compared to those in the high baseline group after the vaccination irrespective of the treatment (**Table 2**). The seroconversion rate comparisons without stratification for all the vaccine strains have been reported elsewhere (44). The age distribution was statistically similar across the MAVRICS study participants in the high and low A/Victoria HAI baseline groups (**Supplementary Figure S2**). Next, we analyzed the correlation between the pre-existing levels and the subsequent post-vaccination fold-change of each analyte stratified by the baseline (**Supplementary Figures S3**, **S4**). The following formula was used to calculate the vaccine induced fold change: (post-vaccination levels-pre-vaccination levels)/post-vaccination levels. This data analysis strategy facilitated the identification of the immune factors exclusively modulated by the melatonin treatment within high and low baseline individuals.

**Table 1:** HAI Seroconversion rates (95% CIs) of the melatonin recipients and control vaccinees stratified by the baseline HAI titers of the A/Victoria (H1N1) strain.

Seroconversion rate (95% CI) (%)			
A/Victoria high HAI baseline			
Influenza strain	Melatonin (n=22) (%)	Control (n=23) (%)	P value
A/Victoria	4.55 (0.23, 21.80)	4.35 (0.22, 20.99)	>0.9
A/Victoria low HAI baseline			
Influenza strain	Melatonin (n=11) (%)	Control (n=12) (%)	P value
A/Victoria	25 (8.89, 53.2)	27.27 (9.74, 56.5)	>0.9
Statistical comparison of seroconversion rates between high and low HAI baseline participants within each cohort			
Influenza strain	Melatonin (P value)	Control (P value)	
A/Victoria	0.11	0.08	

Seroconversion rates were defined as the percent of subjects who reached a post-vaccination titer ≥1:40 with a pre-vaccination titer <1:10, or who had a four-fold rise or higher post-vaccination titer with a pre-vaccination titer ≥1:10 (44). Table shows seroconversion rates at 95% confidence intervals (CI) (lower and upper limits within brackets) and *P* values for each comparison (Fisher’s exact test, two-sided alpha = 0.05).

**Table 2:** Mean fold increase of HAI antibody titers after seasonal influenza vaccination stratified by the baseline HAI titers of the A/Victoria (H1N1) strain.

HAI (GMT) Titer mean fold increase in melatonin recipients			
Influenza strain	High baseline (n=22)	Low baseline (n=11)	P value
A/Victoria	1.33	5.62	0.027*
HAI (GMT) Titer mean fold increase in control vaccinees			
Influenza strain	High baseline (n=23)	Low baseline (n=12)	P value
A/Victoria	1.3	8.3	0.004**

P values for each comparison (Mann Whitney test, two-sided alpha = 0.05). *P < 0.05; **P < 0.01.

#### 3.1.1 Immune factors solely modulated by the exogenous melatonin treatment in the A/Victoria high HAI baseline group

**Figure 3:**
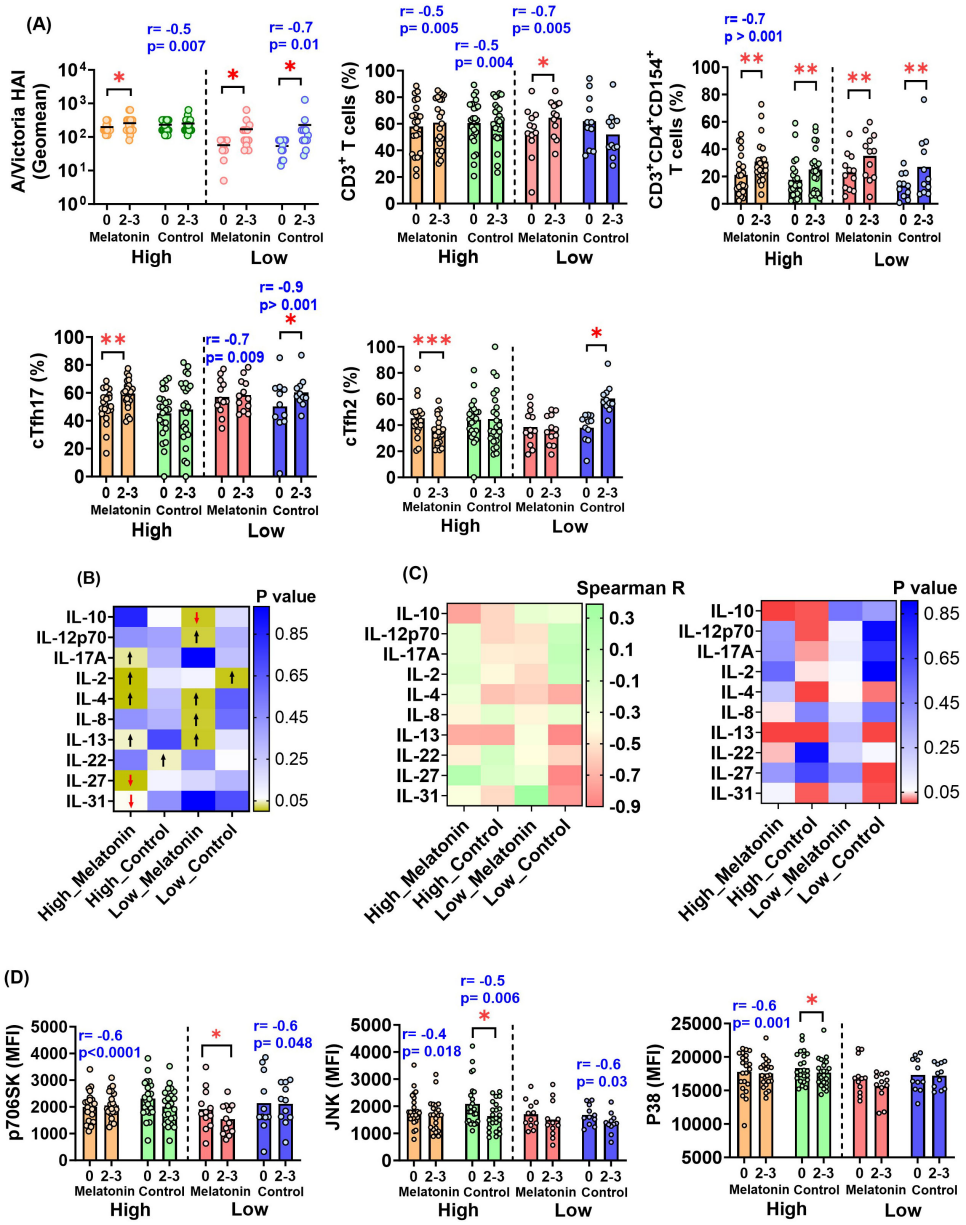
Immune factors modulated by exogenous melatonin in the individuals with high and low HAI baseline responses against A/Victoria strain. **(A)** A/Victoria specific HAI titers and HA antigen-specific T cell responses in the melatonin recipients and the control vaccinees based on the baseline HAI responses against A/Victoria strain before (0) and after (2-3) the vaccination. **(B)** Distinct HA antigen-specific cytokine responses in the high and low HAI baseline melatonin recipients and control vaccinees. The heatmap visualizes the p-values generated by comparing pre- and post-vaccination responses for each cytokine. The arrows represent a significant increase (black upward arrow) or a significant decrease (red downward arrow) after the vaccination. **(C)** Spearman R heatmap (left panel) illustrating the correlation coefficient (r) between pre-existing titers and the post-vaccination fold-change of HA antigen-specific cytokines in the high and low HAI baseline melatonin recipients and control vaccinees. The p-value heat map (right panel) summarizes the p-values of the respective correlations. HA antigen-specific cell signaling factors in the high and low HAI baseline melatonin recipients and the control vaccinees before (0) and after (2-3) the vaccination. MFI- Mean Fluorescent Intensity. For **(A, D)** the two baseline groups are divided by a dotted line in each plot. The mean value for each cohort is represented by a short, dashed line on the scatter plots. Asterisks (*) denote significant differences between pre- and post-vaccination at 0.05 alpha level. *P < 0.05; **P < 0.01; ***P < 0.001. The Spearman R correlation coefficient (r) and p values shown in blue indicate a significant correlation between pre-existing levels and the post-vaccination fold-change of a given analyte within each respective sub-cohort. Paired comparisons were carried out using either paired sample T test or the Wilcoxon matched pairs signed rank test after testing for normality. High and Low in the X axis represents A/Victoria high HAI baseline and A/Victoria low HAI baseline cohorts respectively. Melatonin high HAI baseline, *n=22*; Control high HAI baseline, *n=23*; Melatonin low HAI baseline, *n=12*; Control low HAI baseline, *n=11*.

We identified several immune factors modulated solely by the exogenous melatonin treatment in the high baseline group following vaccination (**Figures 3A, B**). High baseline melatonin recipients showed a significant increase in A/Victoria HAI titers, antigen-specific cTfh17 frequency (**Figure 3A**) and cytokines; IL-2, IL-4 and IL-17A (**Figure 3B**, **Supplementary Figure S5**) and a significant decrease in cTfh2 (**Figure 3A**), IL-27 and IL-31 levels (**Figure 3B**, **Supplementary Figure S5**) at 2–3 weeks post-vaccination. In fact, vaccine induced fold-changes in the above immune factors were not significantly correlated to their pre-existing levels, suggesting that the melatonin alone may drive these changes (**Figures 3A, C**, **Supplementary Figure S3A**). We also found significantly higher post-vaccination levels of antigen-specific IL-13 in the high baseline melatonin recipients (**Figure 3B**, **Supplementary Figure S5**), yet those changes were negatively influenced by pre-existing IL-13 levels (**Figure 3C**, **Supplementary Figure S3A**). Vaccination alone had no significant impact on A/Victoria HAI titers and antigen-specific cTfh17, cTfh2, IL-2, IL-4, IL-17A, IL-27, IL-31 and IL-13 levels in the high baseline group (**Figures 3A, B**, **Supplementary Figure S5**). However, there was a strong negative correlation between the pre-existing titers of A/Victoria HAI antibodies, IL-2, IL-4, IL-17A, IL-31, IL-13 antigen-specific cytokines and the corresponding post-vaccination fold-changes in the high baseline control vaccinees (**Figures 3A, C**, **Supplementary Figure S3B**).

#### 3.1.2 Immune factors solely modulated by the exogenous melatonin treatment in the A/Victoria low HAI baseline group

Interestingly, all the immune factors significantly and exclusively influenced by melatonin in the low baseline group were different from those of the high baseline group except for IL-4 and IL-13 (**Figures 3A, B, D**, **Supplementary Figure S5**). Melatonin recipients who belonged to the low baseline group showed a significant increase in antigen-specific CD3^+^ T cells (**Figure 3A**), IL-4, IL-8, IL-12p70 and IL-13 cytokines following vaccination (**Figure 3B**, **Supplementary Figure S5**). In contrast, melatonin treatment induced a significant reduction in the regulatory cytokine IL-10 (**Figure 3B**, **Supplementary Figure S5**) and the cell signaling factor p706SK in the low baseline group (**Figure 3D**). Except CD3^+^ T and cTfh17 cell frequency, vaccine induced fold-changes in the other factors were not significantly correlated to their pre-existing levels in the low baseline melatonin recipients (**Figures 3A, C**, **Supplementary Figure S4A**). The low baseline control vaccine recipients did not exhibit significant changes in any of the aforementioned immune factors (CD3^+^ T cells, IL-4, IL-8, IL-12p70, IL-10 and IL-13) (**Figures 3A, B**, **Supplementary Figure S5**). Notably, there was a significant increase in both cTfh17 and cTfh2 cell subsets following vaccination in the low HAI baseline control group (**Figure 3A**), where no such changes were observed in their melatonin counterparts (**Figure 3A** **).** The fold-change in IL-4, IL-13 and p70S6K expression was negatively correlated with pre-vaccination titers in the low baseline control vaccinees (**Figures 3C, D**, **Supplementary Figure S4B**).

#### 3.1.3 Immune factors impacted by the seasonal influenza vaccination alone

We next focused on the immune factors particularly governed by the seasonal influenza vaccination alone independent of the melatonin treatment (**Figure 3A**). Therefore, any factor displaying significant changes in both groups of vaccinees was attributed the effects of the vaccination itself. To our surprise, in the high baseline group, antigen-specific, activated CD3^+^CD4^+^CD154^+^ T cells were the only factor significantly affected by the vaccination alone, leading to an increase in both melatonin recipients and controls (**Figure 3A**). Likewise, both groups of vaccine recipients in the low baseline group also showed increased levels of activated CD3^+^CD4^+^CD154^+^ T cells (**Figure 3A**). Interestingly, contrary to the high baseline group, every individual in the low baseline group had significantly elevated A/Victoria HAI titers, independent of the melatonin treatment (**Figure 3A**). In addition, there were several immune factors in both high (IL-22, JNK and P38 cell signaling factors) and low (cTfh17 and cTfh2 frequency, IL-2 levels) baseline groups, which displayed changes only in the control vaccinees, but not in the melatonin recipients (**Figures 3A, B, D**). It is more likely that the exogenous melatonin may have stabilized or reversed the immunological changes induced in the control vaccinees by the IIV4 vaccination. However, factors such as JNK and P38 in high baseline melatonin recipients (**Figure 3D**, **Supplementary Figure S3A**) and cTfh17 levels in low baseline vaccine recipients (melatonin and control) (**Figure 3A**, **Supplementary Figures S4A, B**) were also affected by their pre-vaccination levels, given the strong negative association with the post-vaccination fold-change.

#### 3.1.4 Overview of the key immune factors modulated by melatonin treatment in high and low baseline groups

**Figure 4:**
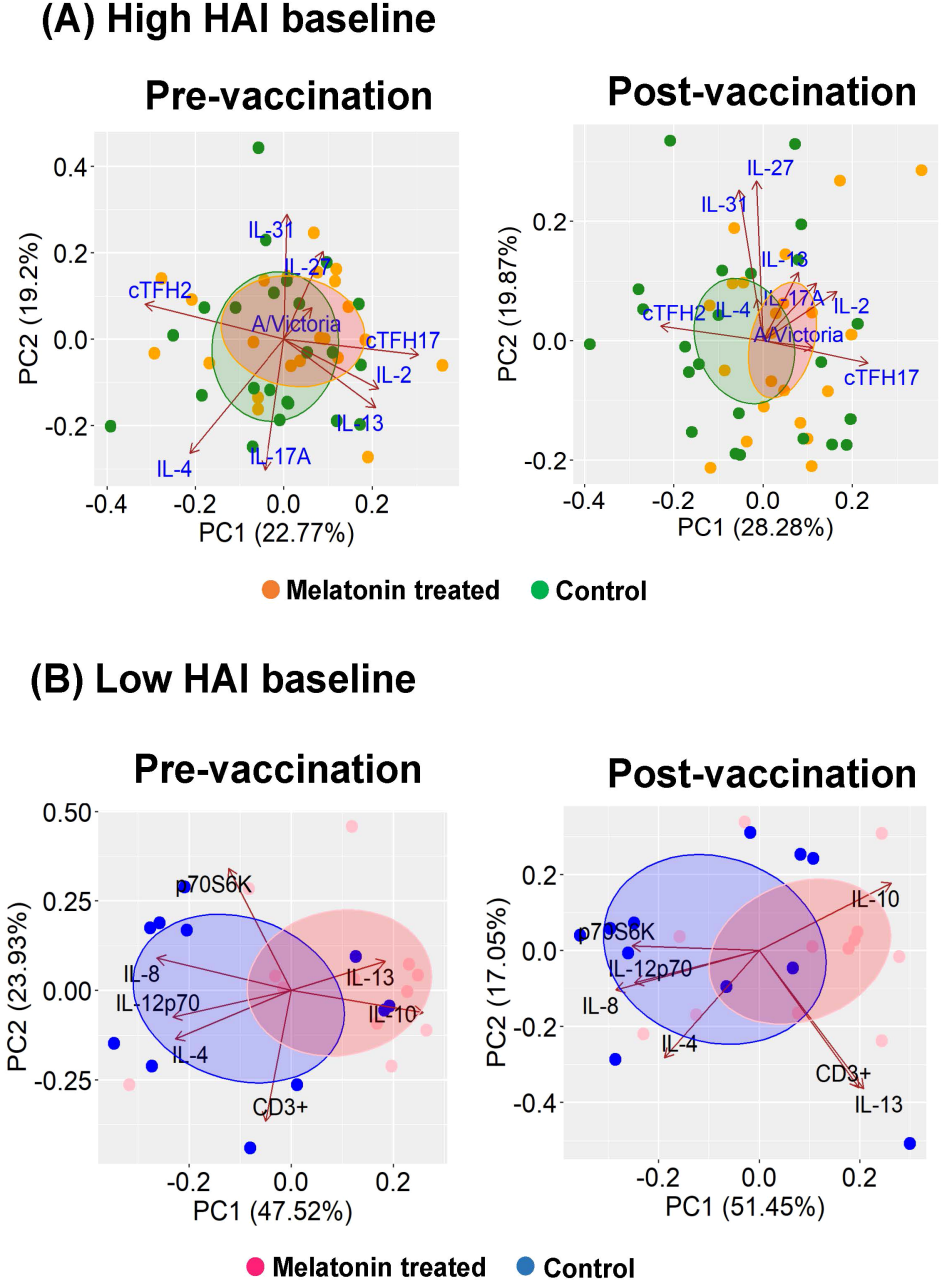
Key contributors of the melatonin mediated immunomodulation in A/Victoria HAI high and low baseline groups. Key immune factors modulated by melatonin administration were identified by performing principal component analyses (PCA) for the **(A)** high baseline group and **(B)** low baseline group. Dots represent individuals in respective treatment group melatonin *vs*. vaccine only (control) group. Loading vectors represent immune factors solely impacted by the melatonin treatment. The direction and the length of the vectors indicate the magnitude of contribution to the respective profile. Concentration ellipses enclose data points within each group. Melatonin high HAI baseline, *n=22*; Control high HAI baseline, *n=23*; Melatonin low HAI baseline, *n=12*; Control low HAI baseline, *n=11*.

The principal component analysis (PCA) plots in **Figure 4** visualize the key immune factors modulated by the melatonin in the high (**Figure 4A**) and low (**Figure 4B**) baseline groups. The shift in the immune responses in the melatonin recipients after the vaccination in the high baseline group was dominated by the cTfh17 levels, closely associated with the A/Victoria HAI titers (**Figure 4A**, right panel: Post-vaccination). Also, PCA plots further identified two additional cytokine clusters, defined by IL-2, IL-17A, IL-13 and IL-4, IL-27, IL-31; shown to be responsible for vaccine induced variations in the high baseline melatonin recipients (**Figure 4A**, right panel: Post-vaccination). The melatonin recipients and the control vaccinees in the low baseline group seemingly belonged to two separate clusters even before the vaccination (**Figure 4B**, left panel: Pre**-**vaccination). Yet, melatonin in fact, added an additional level of variability (variance in PC1 increased from pre- 47.2% to post-vaccination – 51.45%) (**Figure 4B**: Pre- vs post-vaccination) and induced a more coordinated immune response after the vaccination. This shift in the immune landscape was characterized by positively associated CD3^+^ T cells and IL-13 levels as well as negatively associated IL-10 and IL-4 levels (**Figure 4B**, right panel: Post-vaccination).

### 3.2 Melatonin treatment does not impact the global cytokine profile induced by influenza vaccination

**Figure 5:**
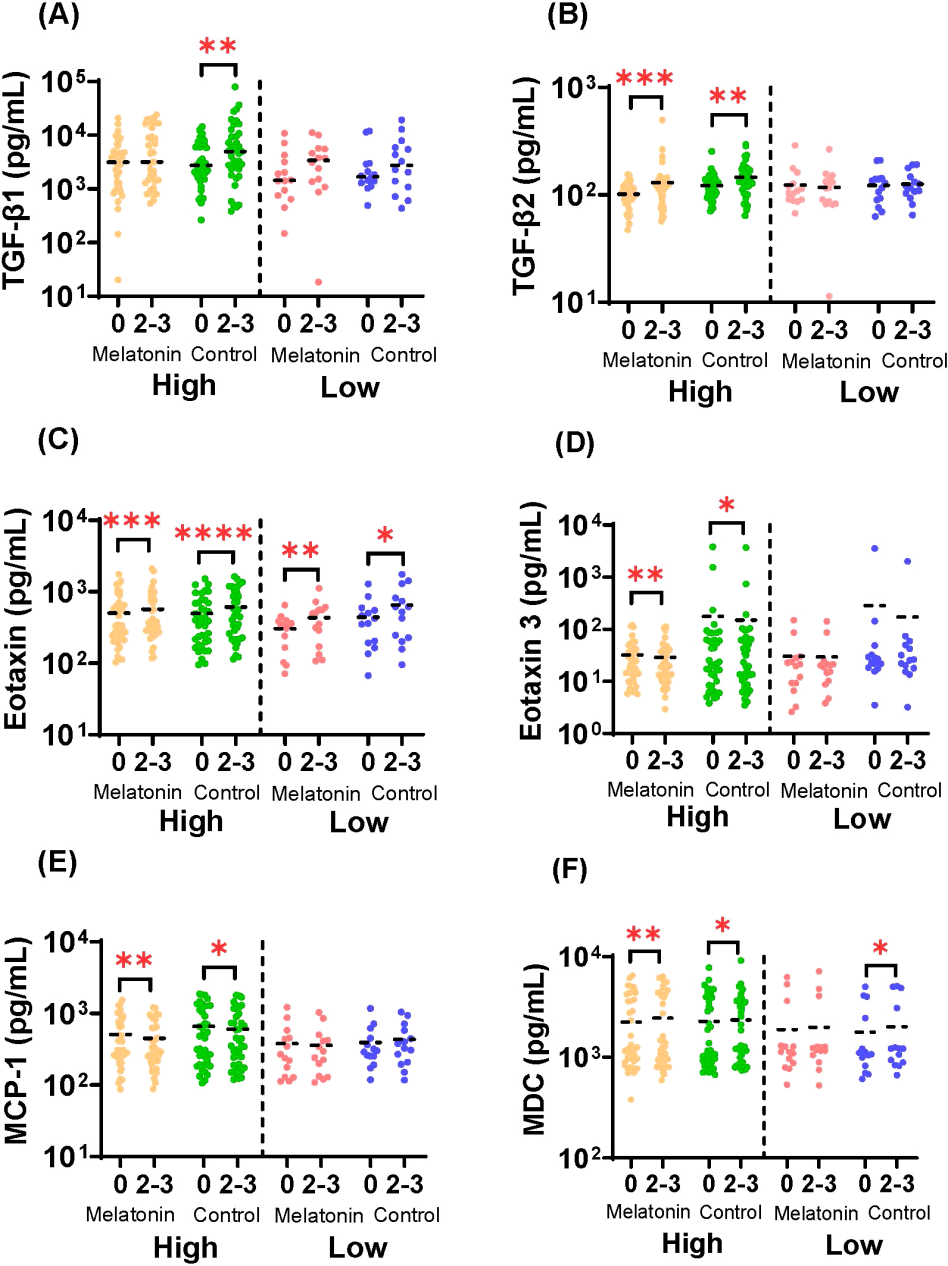
Serum cytokine and chemokine responses. Concentrations of **(A)** TGF-β1 **(B)** TGF-β2 **(C)** Eotaxin **(D)** Eotaxin-3 **(E)** MCP-1 and **(F)** MDC in sera of melatonin recipients and control vaccinees stratified by baseline HAI responses against A/Victoria strain before (0) and after (2-3) the vaccination. High and Low in the X axis represents A/Victoria high HAI baseline and A/Victoria low HAI baseline cohorts respectively. The dashed line represents the mean value for each cohort. Paired comparisons were carried out using either paired sample T test or the Wilcoxon matched pairs signed rank test after testing for normality. Asterisks (*) denote significant differences between pre- and post-vaccination at 0.05 alpha level. *P < 0.05; **P < 0.01; ***P < 0.001; ****P < 0.0001. Melatonin high HAI baseline, *n=39*; Control high HAI baseline, *n=39*; Melatonin low HAI baseline, *n=14*; Control low HAI baseline, *n=14*.

Changes in the circulating cytokine and chemokine responses in humans after the influenza vaccination have been well documented (59, 60). Therefore, we also evaluated the melatonin mediated modulation of the global cytokine landscape in the melatonin recipients compared to the control group. Here we used a customized mesoscale assay to analyze the circulating levels of serum cytokines and chemokines in the melatonin treated and control vaccinees in the high and low baseline groups. **Figure 5** shows the analytes that were significantly changed 2–3 weeks after the vaccination. Interestingly, except for TGF-β1, the significant changes seen in TGF-β2, Eotaxin, Eotaxin-3, MCP-1 and MDC were induced by both groups of vaccinees with the high baseline levels, independent of the melatonin-treatment. While the TGF-β1 levels were significantly increased in the high baseline-control vaccinees, melatonin treatment appeared to mitigate this effect by stabilizing TGF-β1 levels to remain relatively unchanged (**Figure 5A**). Similarly, melatonin mediated stabilizing effect was also observed with MDC levels in the low baseline group (**Figure 5F**), even though it was not so profound compared to TGF-β1 (**Figure 5A**). Hence, our data show that the global cytokine profile induced by the IIV4 vaccination largely remained unaffected by the exogenous melatonin. In addition, IIV4 vaccination selectively altered the cytokines and chemokines primarily in the high baseline group (**Figures 5A, B, D, E**), while Eotaxin was the only analyte substantially changed across all the vaccinees irrespective of the baseline HAI titers (**Figure 5C**).

## 4 Discussion

Our study presents the first clinical evidence supporting the immunomodulatory effect of melatonin in conjunction with a clinically approved vaccine. Here we report that the melatonin could selectively and potentially favorably modulate the antigen specific immune responses induced by the seasonal influenza vaccination without substantially altering the global cytokine profile. Notably, these findings stand in stark contrast to our previously published data (44), attributed to differences in research and data analysis strategies employed in the present study.

The impact of pre-existing immunity on subsequent vaccine responses has been a subject of considerable interest in influenza research. In healthy adults, pre-existing influenza antibodies can significantly reshape both the quality, and the magnitude of humoral immune response induced by seasonal IIVs (56, 61). When present in high levels, pre-existing influenza antibodies often diminish the immune responses to subsequent vaccinations (56, 62–65). This could be attributed to several potential mechanisms that may disrupt efficient activation of antigen specific memory B cell pools (65). For instance, pre-existing antibodies can form immune complexes with vaccine antigens or inactivated viruses, which can be rapidly cleared by phagocytes through Fc receptor engagement or complement mediated opsonization. In addition, immune complex engagement of Fc receptors expressed on natural killer cells trigger the release of cytotoxic molecules to eliminate such complexes. Both mechanisms could potentially reduce the availability of vaccine delivered antigenic epitopes necessary to activate the B cell memory, dampening the boosting response. Additionally, binding of antibody-antigen immune complexes to the inhibitory Fc receptors expressed on B cells could directly suppress the activation of naïve or memory B cells. Besides, antibody-driven epitope masking could be the most significant contributor for the reduced availability of vaccine delivered antigenic epitopes for efficient B cell recall responses. Immune responses induced by seasonal influenza vaccines are heavily influenced by the “original antigenic sin” or “immune imprinting” established by prior influenza infections or repeated vaccinations (61). As a result, previously encountered immunodominant epitopes generally dictate the pre-existing antibody response (56, 62). When these antibodies encounter the same immunodominant epitopes delivered during the subsequent vaccinations, they could block or mask such epitopes and the neighboring ones (due to steric hindrance). This in turn would make those antigenic epitopes inaccessible by the immune cells preventing a robust memory B cell activation. Moreover, the novel or conserved vaccine epitopes (ex: HA stalk domain) may remain neglected due to the “immune imprinting” by previous exposures. In fact, repeated influenza vaccinations and high levels of pre-existing antibodies seem to negatively impact the antibody affinity maturation process and HA stalk targeted broadly neutralizing antibody responses (56, 62, 65, 66). Our data suggest that the use of immunomodulators such as melatonin may selectively disrupt this interplay specifically in the adults with high pre-existing titers against the homologous vaccine strains. When the pre-existing HAI antibodies were high, melatonin appeared to change the post-vaccination HAI antibody landscape at an individual level reflected only by the significant differences in pre- and post-vaccination antibody titers. Yet, the predictors of overall post-vaccination HAI responses such as antibody fold-rise and seroconversion rates remained low in those individuals. Although the exact mechanisms remain unclear, melatonin is more likely to modulate the affinity and the epitope repertoire of the antigen-specific antibodies, without affecting the overall post-vaccination antibody response. It has been previously suggested that quantity and the specificity of the antibody epitope repertoire may impact the overall outcome of the influenza immunity (62). As a naturally produced immunomodulator, melatonin may be inherently selective towards the target rather than to induce broader or global changes in the immune system as indicated by largely unchanged global cytokine profile in the melatonin recipients.

Tfh cells are pivotal in generating and maintaining a robust humoral immunity. Tfh cells promote B cell activation and differentiation, contributing to the formation of memory B cells. Circulating counterparts of Tfh (cTfh) cells expressing CD4^+^CXCR5^+^ are divided into three different subtypes with distinct functions and phenotypes defined by the presence of chemokine markers CXCR3 and CCR6 (50, 67–69). The data suggest that melatonin has a selective modulatory effect on the antigen-specific cTfh subset response based on the levels of pre-existing HAI antibodies and the previously imprinted immune landscape. Our data represent the collective cTfh responses against the four HA antigens (representing the four vaccine strains), stratified by the baseline levels of A/Victoria HAI antibodies. Melatonin clearly favors a cTfh17 (CXCR3^-^CCR6^+^) dominant response when the pre-existing antibody levels are high, as evident by an increase in cTfh17 and a parallel decrease in cTfh2 (CXCR3^-^CCR6^-^) levels post-vaccination. The data need to be interpreted with caution for the low HAI baseline melatonin recipients as they already had relatively high pre-vaccination cTfh17 levels (**Figure 3A**, melatonin treated: pre-vaccination cTfh17 mean frequency; 50.50% in high baseline and 57.12% in low baseline). In fact, melatonin seems to facilitate the maintenance of cTfh17 levels at a maximum threshold counteracting the negative effect of the pre-existing cTfh17 levels in the low baseline individuals. As a result, melatonin tends to indirectly promote the cTfh17 response, where it remained unchanged and present at consistently high levels following vaccination. Unlike the melatonin recipients, the control IIV4 vaccinees show no dominance between post-vaccination cTfh17 and cTfh2 responses irrespective of the baseline antibody levels (**Figure 3A**). Seemingly, the seasonal vaccination itself can induce substantial levels of cTfh17 and cTfh2 subsets. Particularly, in the absence of a significant baseline immune imprinting, IIV4 vaccination favors a balance between cTfh17 and cTfh2 subsets in the low baseline participants. Retrospectively, the vaccine itself could not override the previously established immune landscape, as indicated by the lack of boosting responses in HAI antibodies and cTfh subsets in the high HAI baseline group. Effector response (day 7–14 after vaccination) to trivalent influenza vaccination has been shown to be dominated by cTfh1 like subsets (70, 71), whereas cTfh17 found to be dominated in the memory phase (71). Clearly, melatonin could alter the cTfh dynamics during the effector phase by selectively favoring towards a cTfh17 dominated response, which may be the key to override the pre-existing immune landscape imprinted by previous influenza exposures. Both cTfh17 and cTfh2 subsets are efficient helpers in naïve B cell proliferation, plasma cell differentiation and IgG antibody class switching (68, 69). However, the two subsets appear to have a differential IgE class switching profile, where cTfh17 does not seem to induce IgE production from naïve B cells (68). Also, vaccine specific cTfh17 cells are believed to be superior to cTfh1 and cTfh2 cells in Tfh memory maintenance (71). Superior immunological memory maintenance of Tfh17 like cells could be attributed to their better survival capacity and better maintenance of the potential to differentiate into germinal center Tfh cells *in vitro* (71). In fact, Zaire Ebolavirus (ZEBOV) glycoprotein specific cTfh17 have been shown to be associated with persistent antibody responses after 56 days post rNSV-ZEBOV vaccination (46). A favorable IgG class switching ability equipped with better memory maintenance may help cTfh17 cells to promote antibody diversity and affinity maturation to mitigate phenomena like antibody-driven epitope masking. The clinical relevance of melatonin on long-term B cell memory maintenance and protection against homologous and heterologous influenza strains requires further investigation, particularly in adults with considerably complex pre-existing immunity to influenza and underlying disease conditions.

Melatonin may selectively promote an optimal cytokine environment for cTfh17 cell activation, antibody production and memory B cell development to potentially mitigate the challenges of pre-existing immunity. Overall, pro-inflammatory cytokines including IFN-γ, TNF-α, IL-1β, IL-1α, IL-6, IL-8, IL-27, IL-31, IL-17A and IL-17F remained at moderate to high levels among both melatonin and control vaccine recipients before and after the vaccination (**Supplementary Figure S5**). For the final analysis, we only selected the antigen-specific cytokines significantly changed after the vaccination within each sub cohort stratified by the baseline. In the high HAI baseline melatonin recipients, the post-vaccination responses were characterized by elevated levels of IL-17A, IL-2, IL-4, IL-13 and decreased levels of IL-27 and IL-31. The melatonin recipients in the low HAI baseline group, had decreased levels of IL-10, and increased levels of IL-4, IL-13, IL-8 and IL-12p70. The observed increase of IL-17A aligns with the increased cTfh17 responses in the high baseline individuals, as IL-17 is predominantly secreted by cTfh17 cells (50, 68, 69). While IL-21 is traditionally regarded as the key Tfh cytokine facilitating B cell help (69), pre-clinical studies have suggested that IL-17 can also promote germinal center development, antibody class switching and B cell differentiation into plasma cells (72). Decreased IL-27 may be linked to its dual, anti- and pro-inflammatory roles mediated through increased expression of c-Maf on CD4^+^ T cells (73). c-Maf signaling promotes IL-10 production, while also supporting cTfh responses by inducing IL-21 production (73). When the baseline immune imprinting is profoundly high, melatonin appears to lower the IL-27 levels, likely modulating IL-10 production to balance anti-inflammatory effects. Subsequently, this could explain the lack of increase in IL-21 levels (and remained at very low levels, **Supplementary Figure S5**), which may be functionally compensated by increased IL-17. While, the role of IL-31 remains less clear, it is known to promote the Th2 phenotype (74). Experiments have shown that IL-31 can induce IL-4 and IL-13 secretion from basophils (75). IL-31 also regulates inflammation by targeting mast cells, dendritic cells and macrophages (75). Although IL-31 levels were also low across the IIV4 participants (**Supplementary Figure S5**), a statistically significant reduction of IL-31 in the high baseline melatonin recipients indicates a regulatory role of melatonin, while promoting the cTfh17 cytokine environment. Despite the increase of the two key cTfh2 cytokines, IL-4 and IL-13 (50, 68, 69), in both high and low baseline melatonin recipients, their absolute levels remained low after the vaccination (**Supplementary Figure S5**), potentially due to relatively lower cTfh2 levels compared the cTfh17. A typical Th2 driven IL-4 and IL-13 immune profile is strongly associated with hypersensitivity and allergic responses, which also promotes B cell activation, IgE class switching and enhanced antibody production. Melatonin appears to shift the immune response away from a traditional Tfh2 cytokine profile, establishing a balance between inflammation and immune activation.

In summary, we show that exogenous melatonin administration can selectively enhance HAI antibodies and antigen-specific cTfh17 responses after seasonal influenza vaccination, particularly in individuals with elevated baseline HAI titers who typically exhibit a limited boosting response to vaccination. Hypothetically, melatonin could engage high-affinity melatonin receptors on CD4+ T cells (8), acting as the third signal to activate unprimed T cells in the presence of antigen-presenting cells (39). This direct activation may favor commitment of potentially novel Tfh17 lineages over Th2 or classical Th17 differentiation, establishing an immunological environment permissive for B cell activation and differentiation while unlocking previously unengaged or low-affinity B cell clones. In parallel, melatonin preserved JNK and p38 MAPK activity in high-baseline individuals (downregulated in their control counterparts – **Figure 3D**), the signaling pathways essential for antigen presentation, T cell activation, and cytokine production (76). Notably, melatonin’s immunomodulatory effects were context-dependent rather than systemic, as global cytokine profile remained largely unchanged. Taken together, our data support the potential use of melatonin as a promising immunomodulator for vaccine development against diseases with complex pre-immunity, such as influenza and SARS-CoV-2, though its mechanisms of action still require further investigation and validation.

### 4.1 Limitations and future directions

Outcome of our study may have been impacted by intrinsic factors such as circadian rhythm integrity, sleep quality, age, metabolic status, and genetic polymorphisms in melatonin receptor or cytokine genes. Additionally, histories of prior influenza and other infections and vaccinations represent important confounders, as they shape baseline immunity and influence the magnitude and direction of recall responses (56, 61, 77). In the present study, age distribution was similar across the different cohorts and did not include adults aged 65 or older. Although, the sleep quality had been measured as a part of the original clinical study, the data have not been published yet. Our current data only focuses on antibodies specific to HA (HAI titers), but not Neuraminidase (NA). It would be worthwhile to explore the HA and NA neutralizing antibody titers and antibody epitope repertoire induced by melatonin in the individuals with varying levels of pre-existing immunity. Additionally, the baseline stratification resulted in a limited sample size, necessitating further studies to strengthen the findings. While individuals having received an influenza vaccination within the past 6 months before enrollment were excluded from the clinical study, we do not have information on the history of prior influenza exposures and vaccinations of the study participants. In addition, the history of SARS-CoV-2 infections/vaccinations had not been explored in the original clinical study, where the virus itself can add immunomodulatory effects to the existing immune landscape.

Unlike clinical studies evaluating novel vaccines for rare diseases, influenza vaccination trials present a significantly more complex challenge due to the pre-existing immune landscape. While typical clinical studies address general confounders such as age, sex, chronic health conditions, and socioeconomic and geographic factors through enrollment criteria, influenza studies require a more nuanced understanding of participants’ immunological history. This includes accounting for repeated influenza infections and vaccinations, as well as prior exposures to other infectious diseases and routine vaccinations. Furthermore, when a study design incorporates an additional treatment component like melatonin, a potent immunomodulator and key regulator of the human circadian rhythm, the timing of vaccination and sampling becomes critically important. Previous research has shown that circadian rhythms could potentially modulate temporal dynamics of serum cytokines (78), various immune cells (79), antibody and T cell responses to vaccinations (80–83). Data also suggest that circadian rhythms may influence influenza antibody responses particularly in older adults (65+) (81, 82). However, inconsistencies, limited sample sizes, and methodological limitations across most of the clinical studies prevent definitive conclusions and necessitate further investigation (84). These factors underscore the need for careful consideration of these variables in future studies, as failure to account for them could compromise the accuracy and generalizability of the findings.

Future research could explore several avenues, including direct receptor-mediated interactions between melatonin and CD4+ T cells; the impact of melatonin on Tfh subset differentiation in both primed and unprimed T cells; and the subsequent effect on B cell differentiation, maturation, and clonal diversity, using both *in vitro* and *in vivo* approaches. Further studies could also investigate the downstream cell signaling pathways involved in these processes. Strategies such as extracellular vesicle cargo analysis of clinical samples (serum and PBMCs) may provide additional insight into melatonin-primed biological pathways.

## Data Availability

The original contributions presented in the study are included in the article/**Supplementary Material**. Further inquiries can be directed to the corresponding author.

## Author disclaimer

Authors are/were military service members or federal/contracted employees of the US Government at the time the study was conducted. Title 17 USC § 105 provides that ‘Copyright protection under this title is not available for any work of the US Government.’ Title 17 USC § 105 defines a US Government work as work prepared by a military service member or employee of the US Government as part of that person’s official duties. The views expressed in this article reflect the results of research conducted by the authors and do not necessarily reflect the official policy or position of the Department of the Navy, Department of Army, Department of Defense, the United States Government, nor Henry M Jackson Foundation for Advancement of Military Medicine, Inc. Material has been reviewed by the Walter Reed Army Institute of Research. There is no objection to its presentation and/or publication. This paper has been approved for public release with unlimited distribution.
