# Supplementary material for: Exogenous melatonin boosts vaccine-induced immunity in individuals with high pre-existing influenza immunity
Source: Front Immunol. 2025 Oct 24;16:1663763. doi: 10.3389/fimmu.2025.1663763 (PMC12592183; doi:10.3389/fimmu.2025.1663763)
Supplement: Supplementary file 1 [file Presentation1.pptx]

## Slide 1
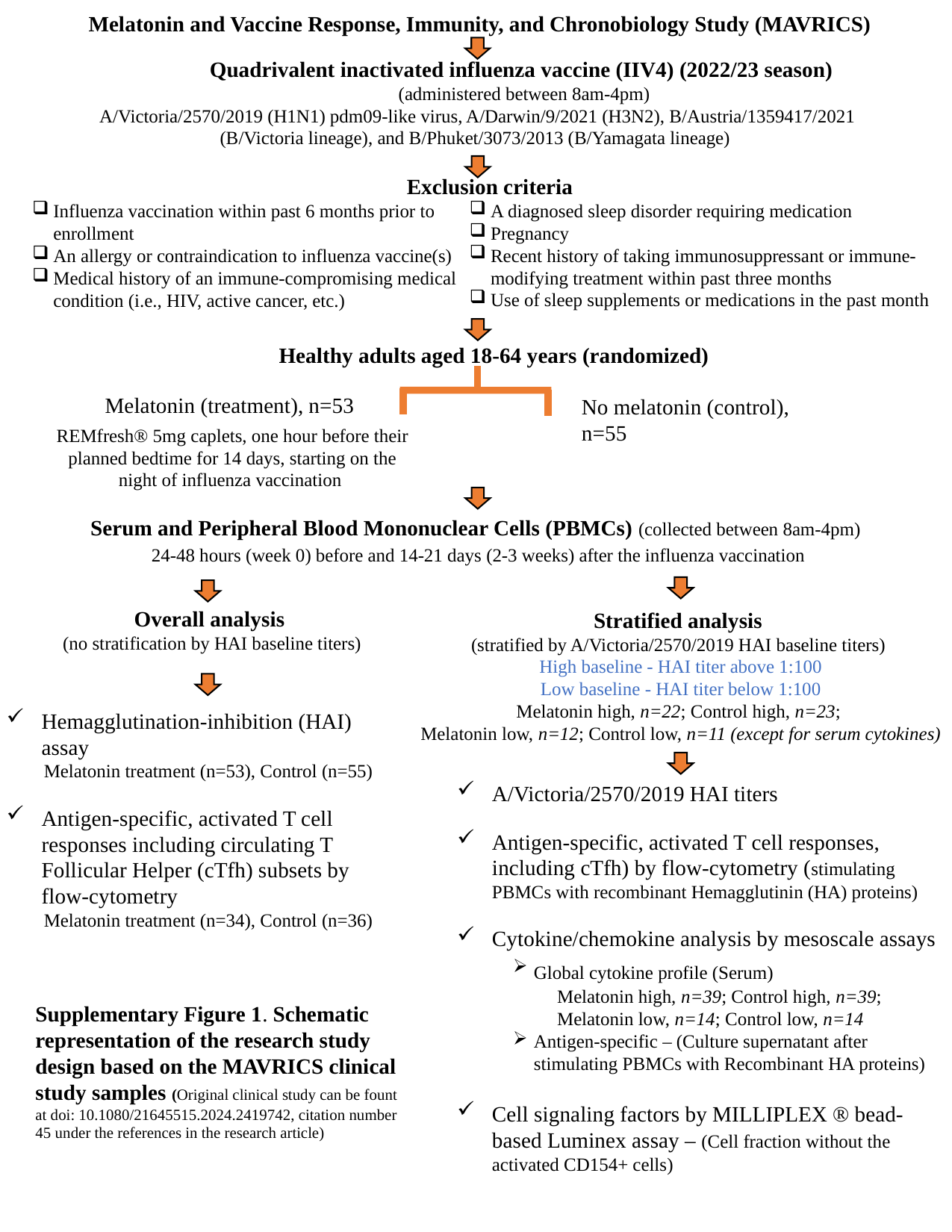

Melatonin and Vaccine Response, Immunity, and Chronobiology Study (MAVRICS)
Quadrivalent inactivated influenza vaccine (IIV4) (2022/23 season)
(administered between 8am-4pm)
A/Victoria/2570/2019 (H1N1) pdm09-like virus, A/Darwin/9/2021 (H3N2), B/Austria/1359417/2021 (B/Victoria lineage), and B/Phuket/3073/2013 (B/Yamagata lineage)
Exclusion criteria
A diagnosed sleep disorder requiring medication
Pregnancy
Recent history of taking immunosuppressant or immune-modifying treatment within past three months
Use of sleep supplements or medications in the past month
Influenza vaccination within past 6 months prior to enrollment
An allergy or contraindication to influenza vaccine(s)
Medical history of an immune-compromising medical condition (i.e., HIV, active cancer, etc.)
Healthy adults aged 18-64 years (randomized)
Melatonin (treatment), n=53
No melatonin (control), n=55
REMfresh® 5mg caplets, one hour before their planned bedtime for 14 days, starting on the night of influenza vaccination
Serum and Peripheral Blood Mononuclear Cells (PBMCs) (collected between 8am-4pm)
 24-48 hours (week 0) before and 14-21 days (2-3 weeks) after the influenza vaccination
Overall analysis
(no stratification by HAI baseline titers)
Stratified analysis
(stratified by A/Victoria/2570/2019 HAI baseline titers)
High baseline - HAI titer above 1:100
Low baseline - HAI titer below 1:100
Melatonin high, n=22; Control high, n=23;
Melatonin low, n=12; Control low, n=11 (except for serum cytokines)
Hemagglutination-inhibition (HAI) assay
 Melatonin treatment (n=53), Control (n=55)
Antigen-specific, activated T cell responses including circulating T Follicular Helper (cTfh) subsets by flow-cytometry
 Melatonin treatment (n=34), Control (n=36)
A/Victoria/2570/2019 HAI titers
Antigen-specific, activated T cell responses, including cTfh) by flow-cytometry (stimulating PBMCs with recombinant Hemagglutinin (HA) proteins)
Cytokine/chemokine analysis by mesoscale assays
Global cytokine profile (Serum)
	Melatonin high, n=39; Control high, n=39; 	Melatonin low, n=14; Control low, n=14
Antigen-specific – (Culture supernatant after stimulating PBMCs with Recombinant HA proteins)
Cell signaling factors by MILLIPLEX ® bead-based Luminex assay – (Cell fraction without the activated CD154+ cells)
Supplementary Figure 1. Schematic representation of the research study design based on the MAVRICS clinical study samples (Original clinical study can be fount at doi: 10.1080/21645515.2024.2419742, citation number 45 under the references in the research article)

## Slide 2
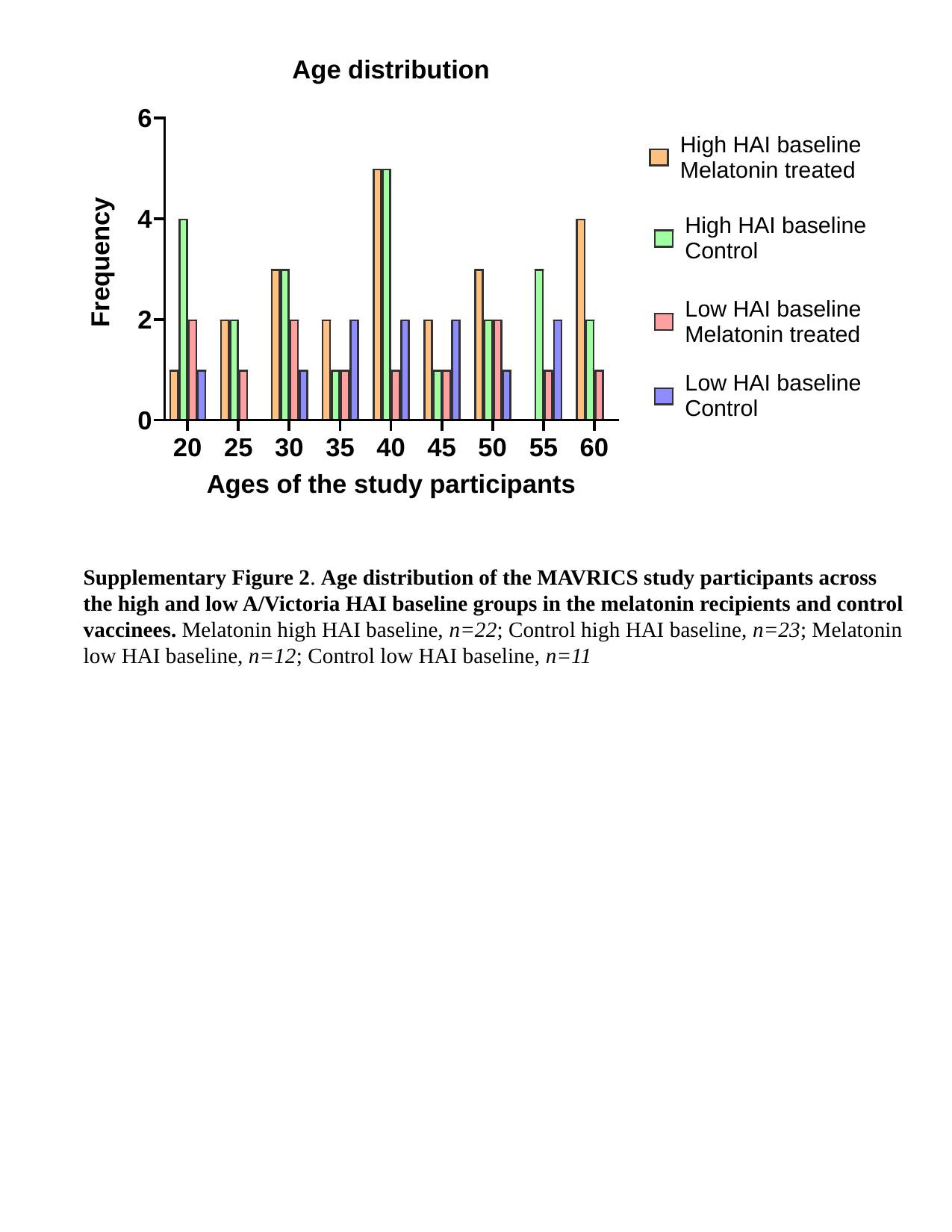

Supplementary Figure 2. Age distribution of the MAVRICS study participants across the high and low A/Victoria HAI baseline groups in the melatonin recipients and control vaccinees. Melatonin high HAI baseline, n=22; Control high HAI baseline, n=23; Melatonin low HAI baseline, n=12; Control low HAI baseline, n=11

## Slide 3
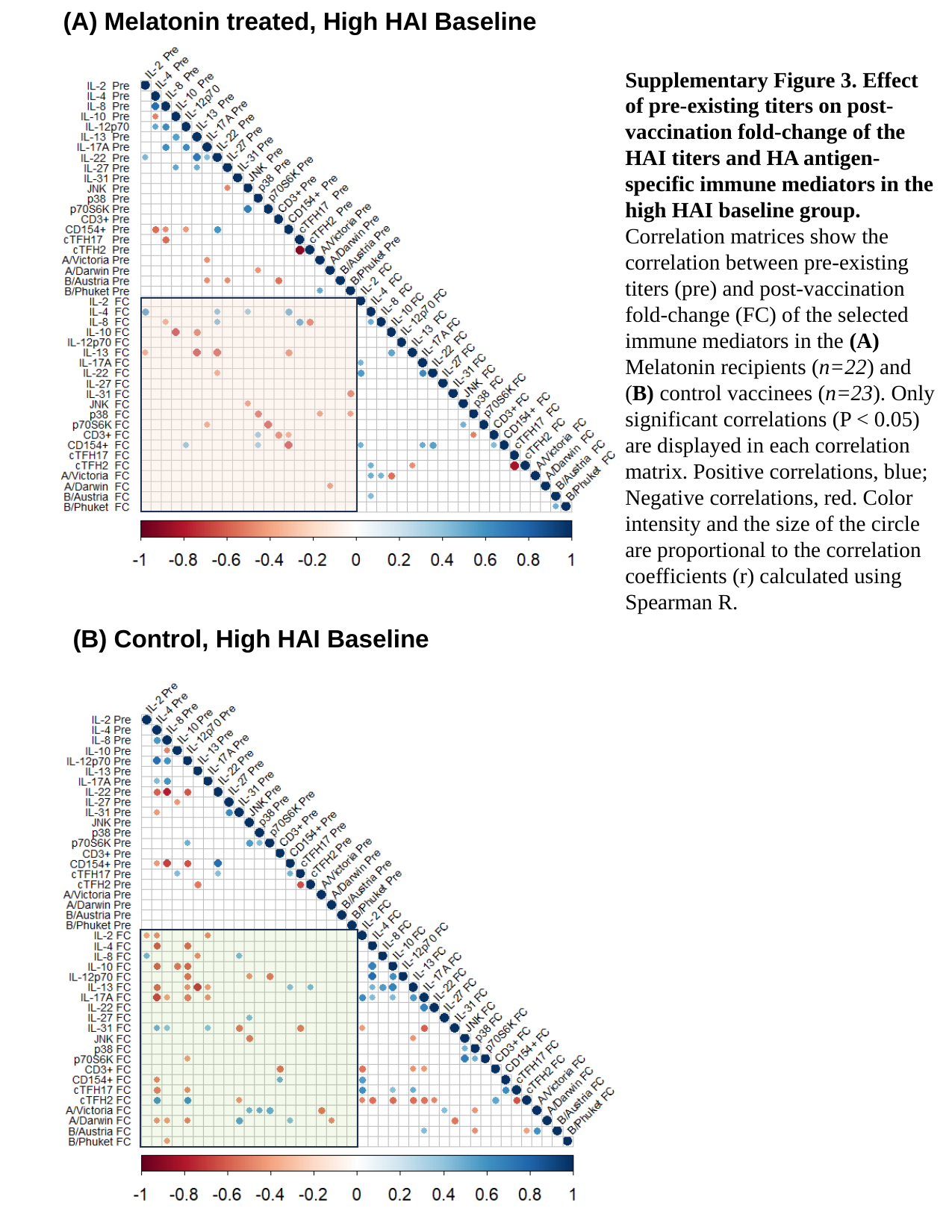

(A) Melatonin treated, High HAI Baseline
Supplementary Figure 3. Effect of pre-existing titers on post-vaccination fold-change of the HAI titers and HA antigen-specific immune mediators in the high HAI baseline group. Correlation matrices show the correlation between pre-existing titers (pre) and post-vaccination fold-change (FC) of the selected immune mediators in the (A) Melatonin recipients (n=22) and (B) control vaccinees (n=23). Only significant correlations (P < 0.05) are displayed in each correlation matrix. Positive correlations, blue; Negative correlations, red. Color intensity and the size of the circle are proportional to the correlation coefficients (r) calculated using Spearman R.
(B) Control, High HAI Baseline

## Slide 4
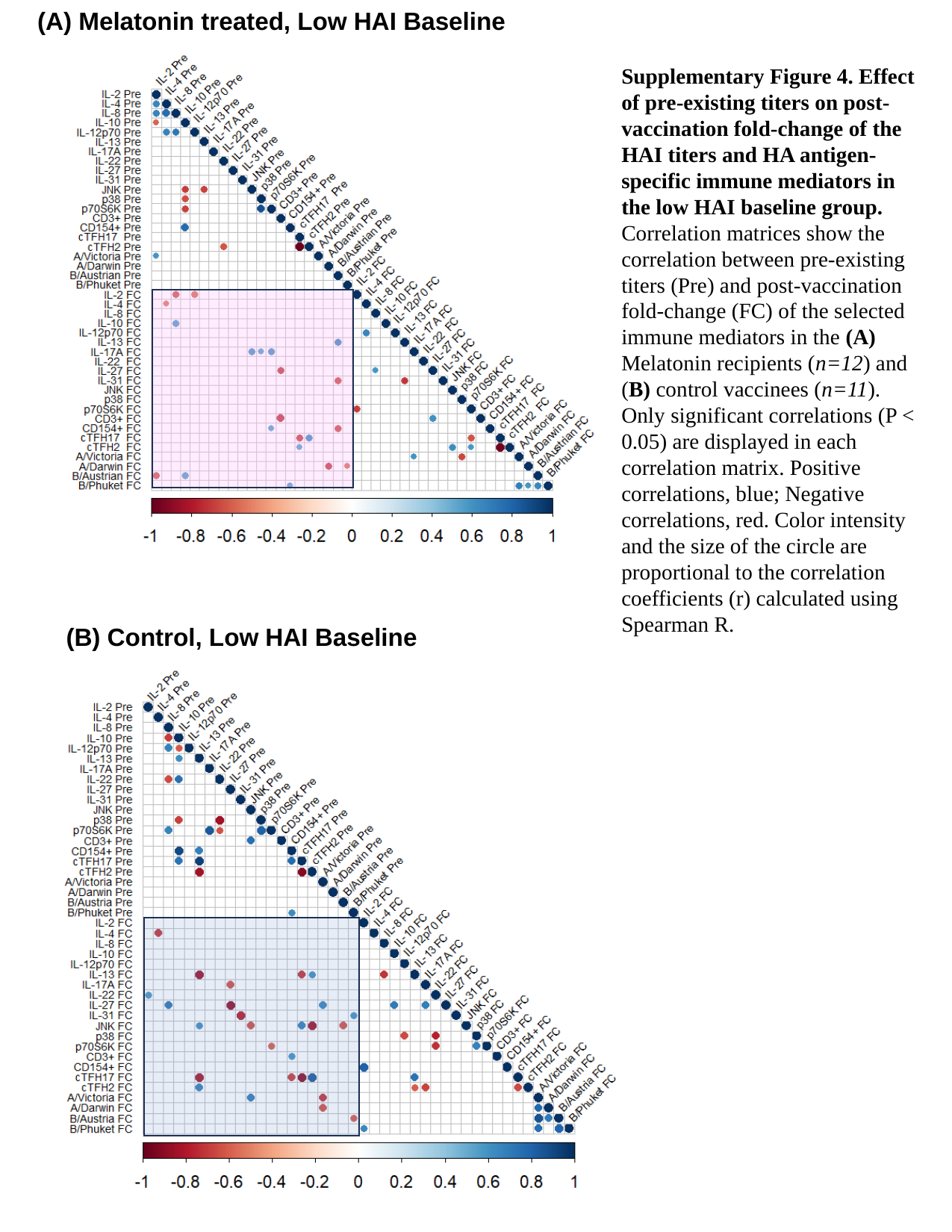

(A) Melatonin treated, Low HAI Baseline
Supplementary Figure 4. Effect of pre-existing titers on post-vaccination fold-change of the HAI titers and HA antigen-specific immune mediators in the low HAI baseline group. Correlation matrices show the correlation between pre-existing titers (Pre) and post-vaccination fold-change (FC) of the selected immune mediators in the (A) Melatonin recipients (n=12) and (B) control vaccinees (n=11). Only significant correlations (P < 0.05) are displayed in each correlation matrix. Positive correlations, blue; Negative correlations, red. Color intensity and the size of the circle are proportional to the correlation coefficients (r) calculated using Spearman R.
(B) Control, Low HAI Baseline

## Slide 5
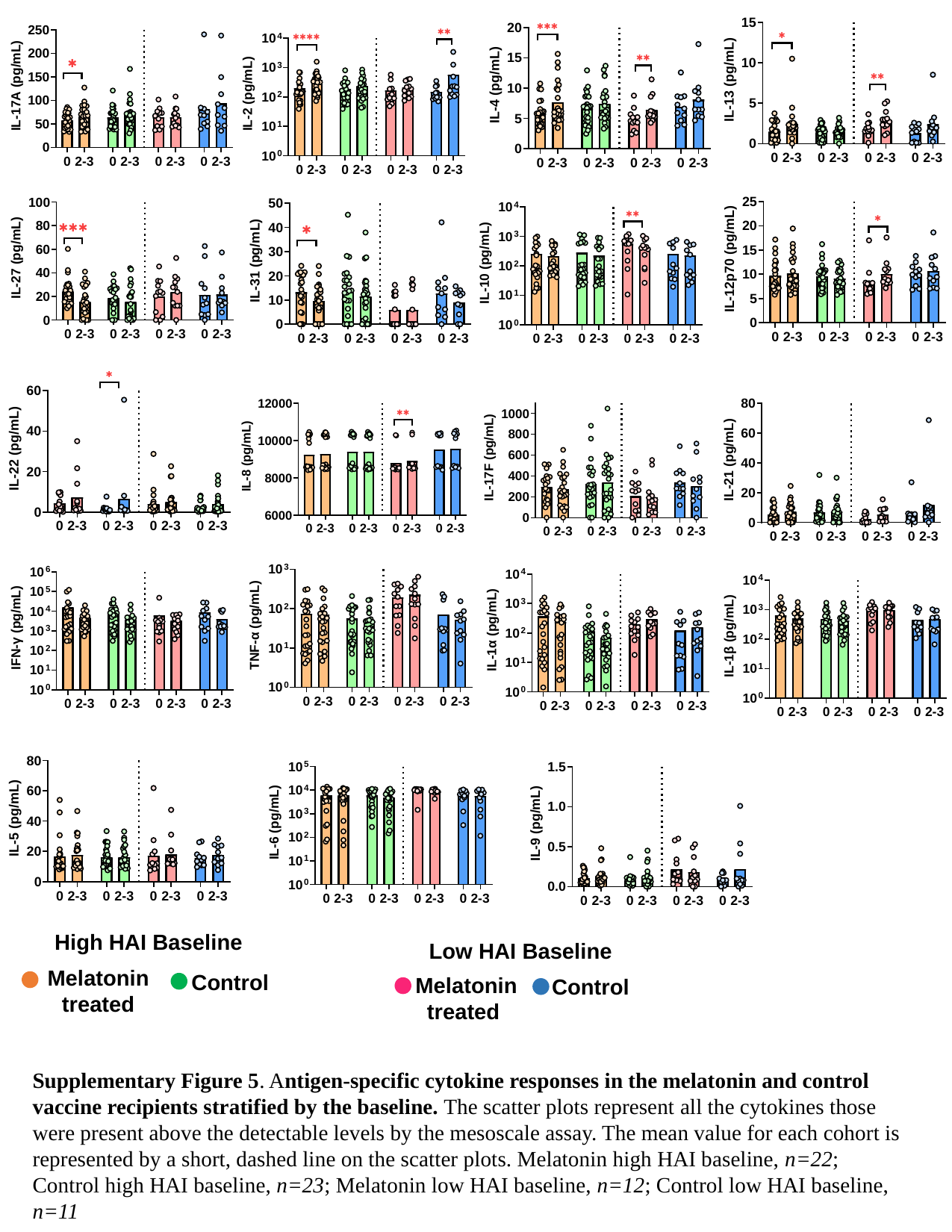

High HAI Baseline
Melatonin treated
Control
Low HAI Baseline
Melatonin treated
Control
Supplementary Figure 5. Antigen-specific cytokine responses in the melatonin and control vaccine recipients stratified by the baseline. The scatter plots represent all the cytokines those were present above the detectable levels by the mesoscale assay. The mean value for each cohort is represented by a short, dashed line on the scatter plots. Melatonin high HAI baseline, n=22; Control high HAI baseline, n=23; Melatonin low HAI baseline, n=12; Control low HAI baseline, n=11
